# Foot‐and‐Mouth Disease Virus Persistence Divergence Within Serotypes and Vaccine Doses in Vaccinated Cattle

**DOI:** 10.1155/tbed/5568178

**Published:** 2026-06-26

**Authors:** Zhihui Zhang, Sumin Wei, Zhidong Teng, Shuanghui Yin, Suyu Mu, Jinwei Huang, Shuang Wang, Guoliang Huang, Yaozhong Ding, Yun Zhang, Yijing Li, Shiqi Sun, Huichen Guo

**Affiliations:** ^1^ State Key Laboratory for Animal Disease Control and Prevention, College of Veterinary Medicine, Lanzhou Veterinary Research Institute, Lanzhou University, Chinese Academy of Agricultural Sciences, Lanzhou, 730046, China, caas.cn; ^2^ College of Veterinary Medicine, Northeast Agricultural University, Harbin, 150030, China, neau.edu.cn; ^3^ College of Veterinary Medicine, Gansu Agricultural University, Lanzhou, 730070, China, gsau.edu.cn; ^4^ School of Life Sciences, Ningxia University, Yinchuan, 750021, China, nxu.edu.cn

## Abstract

Foot‐and‐mouth disease virus (FMDV) persistence in ruminants constitutes the potential risk of disease transmission. While vaccination effectively protects animals against clinical disease, more than 50% of vaccinated cattle can still develop persistent infection. Investigating the potential factors that influence the establishment of FMDV persistence in vaccinated cattle is critical for improving control and eradication strategies in epidemic regions. Here, cattle vaccinated with different doses of serotype O and serotype A vaccines were experimentally challenged with the homologous virus. The overall incidence of the carrier state was 22.5%. Although higher levels and prolonged detection of viral RNA in oesophagal‐pharyngeal fluids (OPFs) were observed in the two higher‐dose groups of both serotypes, neither viral serotype nor vaccine dose significantly influenced the final carrier incidence. Notably, most carriers showed a slower rise in antigen‐specific and neutralizing antibody (NAb) titers postchallenge, resulting in significantly lower antibody titers during the early infection stage, irrespective of the viral serotypes or vaccine doses. Viral genomes and particles were predominantly localized within nasopharyngeal tissues, including the nasopharynx, soft palate, and adjacent lymph nodes and tonsils. In addition, carriers exhibited an increased number of germinal centers in the lymph nodes and tonsils. These findings provide novel insights into the immunological features associated with FMDV persistence in vaccinated cattle and offer a theoretical foundation for optimizing vaccine design and developing targeted immune intervention strategies.

## 1. Introduction

Foot‐and‐mouth disease (FMD) is a highly transmissible viral disease caused by infection with the FMD virus (FMDV), representing a major transboundary economic threat to global cloven‐hoofed livestock industries [1, 2]. While mortality in adult animals is typically low, morbidity in susceptible populations can approach 100%, resulting in considerable economic losses via reduced productivity, regional quarantine practices, and trade barriers [1, 3]. The seven FMDV serotypes (O, A, C, Asia 1, and SAT 1–3) exhibited limited cross‐protection, posing substantial challenges to FMD control and eradication efforts. The prolonged or persistent viral infection within ruminant populations represents a further complicating factor in disease management [4]. Persistent infection, commonly known as the carrier status, is generally defined as the sustained detection of infectious virus in oesophagal‐pharyngeal fluid (OPF) collected by probang cups beyond 28 days after initial viral exposure [5–7]. Studies have shown that approximately 50%–100% of infected cattle, whether vaccinated or not, can develop carriers [8–10], with OPF from carriers being infectious to naive cattle [11–13]. FMDV carriers thus represent a potential long‐term reservoir, increasing the risk of outbreak recurrence and fostering viral evolution that may compromise vaccine efficacy.

FMDV serotypes differ in antigenicity, immunogenicity, transmissibility, and pathogenicity. It has been reported that serotypes SATs and O generally demand higher antigen payloads to achieve equivalent protection compared to serotypes A, C, and Asia 1 [14]. Potency trials conducted at the Pirbright Institute demonstrated that a substantial proportion of animals vaccinated with the serotype SAT2 vaccine developed clinical disease upon challenge, whereas 100% protection was observed in the cohort vaccinated with the serotype A vaccine [15]. Although clinical signs are broadly similar across species, severity varies with viral strain and host susceptibility. Serotypes O and A can spread efficiently among cattle and pigs via direct contact, aerosols, and environmental contamination, often producing pronounced clinical disease. In contrast, serotypes SATs and Asia 1 have narrower transmission ranges and tend to cause milder clinical symptoms [16]. The likelihood and duration of the carrier state are also varied from host species, viral strains, or serotypes. SAT serotypes appear particularly prone to inducing persistence, possibly due to complex immune‐evasion mechanisms [17–19], but are largely confined to sub‐Saharan Africa. Serotypes O and A represent the most prevalent serotypes globally, with both being the primary etiological agents of outbreaks across China [20]. No conclusive evidence exists regarding differences between O and A in their capacity to establish persistent infection, highlighting the need for comparative studies to inform region‐specific control strategies.

Routine vaccination effectively prevents viremia and clinical signs but does not eliminate local infection or reduce the occurrence and duration of persistence [21, 22]. Early studies comparing the incidence of persistent infection in cattle and sheep vaccinated with the O1 Manisa inactivated emergency vaccine or conventional vaccine showed that increasing the vaccine antigen dose induced high neutralizing antibody (NAb) levels more rapidly, thereby suppressing local FMDV replication in the oropharynx, reducing viral shedding in oropharyngeal secretions, and to some extent lowering the proportion of persistently infected animals [23, 24]. However, a study of the A24 Cruzeiro recombinant adenovirus‐vectored vaccine found that, compared with unvaccinated cattle, increasing the antigen dose did not significantly reduce viral shedding or the incidence of persistent infection in vaccinated cattle [5]. These studies further suggest that viral serotype and vaccine dose may influence the occurrence of persistent infection, although systematic comparisons are still lacking.

In this work, we assessed the association of carrier incidence with serotypes or vaccine doses via the evaluation of viral‐shedding patterns and antibody responses in vaccinated cattle. Neither serotypes nor vaccine doses significantly influenced the carrier incidence. Notably, a delayed increase in antibody responses was observed in carriers after challenge, irrespective of the serotypes or vaccine doses. In addition, the virus predominantly persists in nasopharyngeal epithelium and nasopharyngeal‐associated lymphoid tissues, with an increased number of germinal centers within nasopharyngeal‐associated lymph nodes and tonsils in carriers. Our findings contribute to a better understanding of the immunological characteristics of carriers and offer valuable insights for developing optimized vaccination strategies and enhanced FMD control measures in endemic regions.

## 2. Materials and Methods

### 2.1. Virus and Vaccine

FMDV strains utilized in this investigation are the porcine‐origin O/BY/CHA/2010 (JN998085) and bovine‐origin A/HuBWH/CHA/2009 (JF792355.1), which exhibit excellent adaptability in cattle and have been utilized as vaccine strains for decades [25]. The expression, purification, and assembly of FMD VLPs were accomplished according to previously described methods [26]. Briefly, the plasmids of three SUMO fusion proteins (VP0, VP1, and VP3) were transformed into *E. coli* BL21 (DE3) (Stratagen) simultaneously and selected by Amp, Kan, and Chl resistance. The proteins were soluble, expressed at low temperature with 0.3 mM IPTG, and purified by nickel affinity chromatography. Subsequently, they were enzymatically cleaved and self‐assembled into VLPs in vitro. The proteins and VLPs were characterized by SDS‐PAGE, western blotting using a self‐made polyclonal antibody, and dynamic light scattering (DLS) (Figure S1).

### 2.2. Animal Experiment Design and Sample Collection

Forty six‐month‐old cattle‐yaks from Gansu Province, which were prescreened and confirmed to be seronegative for FMDV 3ABC antibody using a competitive ELISA kit (LSBIO), were intramuscularly vaccinated with the virus‐like particle (VLP) vaccines of FMDV A/HuBWH/CHA/2009 and O/BY/CHA/2010 at 35 days before viral challenge. Specifically, 20 cattle received the monovalent O/BY/CHA/2010 VLP vaccine at four graded doses (12.5, 25, 50, 100 μg; n = 5 per dose), while another 20 cattle received the monovalent A/HuBWH/CHA/2009 VLP vaccine at the same four graded doses (12.5, 25, 50, 100 μg; n = 5 per dose). Whole‐blood samples were collected weekly postvaccination for serum isolation. At 35 days postvaccination (dpv), cattle were correspondingly inoculated with 10^4^ infectious doses of FMDV O/BY/2010 or A/HuBWH/CHA/2009 intradermally at six sites on the tongue. To evaluate the efficacy of the viral inoculation, four nonvaccinated cows (two for each serotype) were included.

Clinical symptoms, including vesicles on the feet, oral mucosa, and gums, were monitored every 2 days postchallenge (dpc) until 10 dpc. Samples were collected weekly, including whole blood collected in veterinary disposable collection devices or anticoagulant tubes, OPF collected by probang cups, as well as oral, nasal, and anal swabs placed in tubes containing 1 mL DMEM (Sigma) containing 25 mM HEPES (Sigma). Non‐anticoagulative blood samples and swab samples were centrifuged immediately after collection to obtain serum and mucosal secretions. The probang samples were mixed 1:1 with DMEM containing 25 mM HEPES and thoroughly homogenized. Following centrifugation, the clarified supernatant was collected. All samples were maintained at −70°C pending subsequent procedures.

To assess virus clearance from tissues, cattle were sacrificed at 35 dpc, and a uniform necropsy protocol was performed immediately. Approximately 22 distinct tissues were collected from each animal and cut into 100‐mg portions. Half of the tissue aliquots were rapidly frozen in liquid nitrogen and then maintained at −70°C, while the other half were fixed in 4% formalin and embedded in paraffin until further analysis.

The animal experiments with live FMDV in this study were conducted in the animal biosafety level 3 (ABSL‐3) laboratory of the Lanzhou Veterinary Research Institute, Chinese Academy of Agricultural Sciences. Experimental procedures were authorized by the Gansu Ethical Review Committee and conducted in full compliance with the standards and guidelines of the Gansu Animal Experiments Inspectorate (License Number SYXK‐GAN‐2018‐0005).

### 2.3. FMDV RNA Quantity

RNA was isolated from blood, swabs, OPF, and tissue homogenates employing MiniBEST Viral RNA/DNA Extraction Kit (TaKaRa). Reverse transcription was carried out with PrimeScript RT Master Mix (TaKaRa) following the protocols. FMDV RNA loads were quantified by real‐time PCR targeting the *3D* gene [27]. The obtained cycle threshold (*C*_*T*_) values were converted to RNA copies per μL using a calibration curve generated from 10‐fold sequential dilutions of a recombinant plasmid pET28a‐FMDV/*3D* [28]. Viral load was expressed as log_10_ gene copies/μL. Animals with viral RNA positive and viral recovery in OPF beyond 28 days were defined as FMDV carriers.

### 2.4. Serology Detection

FMDV‐specific total IgG titers were examined using lpELISA Kits (LSBIO) based on the manufacturer’s guidelines.

NAb titers were detected by a microplate neutralization assay. Briefly, the serum samples were incubated at 56°C for 30 min. Serial serum dilutions with two‐fold in DMEM (Sigma) were added to 96‐well plates (50 μL per well) and incubated with the identical volumes of 100 TCID_50_ of infective FMDV O/BY/CHA/2010 or A/HuBWH/CHA/2009 for 1 h at 37°C. BHK‐21 cell suspensions (2.5 × 10^4^ cells per well) were loaded and cultured for 72 h for observation of cytopathic effects. NAb titers were subsequently determined using the Reed and Muench method [29].

### 2.5. Immunohistochemistry (IHC)

After confirming the presence of FMDV RNA in tissues via qRT‐PCR, antigen localization in tissues positive for FMDV RNA was assessed by IHC. Formalin‐fixed paraffin‐embedded tissue blocks were processed into 4 μm‐thick sections, which were subjected to IHC analyses using a self‐made anti‐FMDV 146S antibody according to a standard protocol.

### 2.6. Statistical Analysis

Data analyses and graph generation were accomplished using GraphPad Prism software (v9). Statistical differences between two groups across multiple time points were assessed using multiple *t*‐tests with false discovery rate (FDR) correction (Benjamini and Hochberg method). For comparisons among multiple groups across multiple time points, a mixed‐effects model was used with time and group as fixed factors and subject as a random factor, followed by Tukey’s multiple comparisons test. Data are presented as mean ± standard error of the mean (SEM), with statistical significance defined as *p*‐value < 0.05.

## 3. Results

### 3.1. Infection Dynamics and FMDV Persistence in Vaccinated Cattle

**Figure 1:**
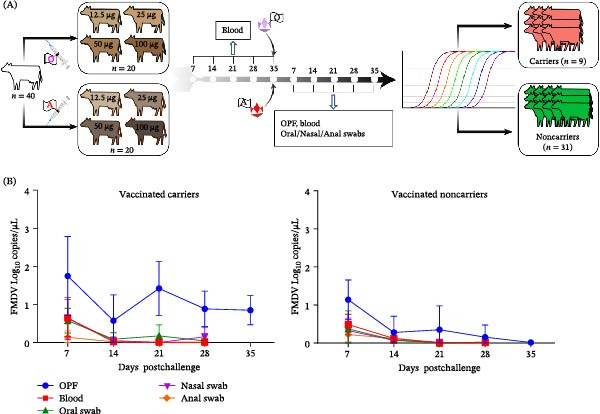
Schematics of the experimental design and FMDV infection dynamics invaccinated cattle. (A) The diagram of the animal experiment design. (B) Detection of FMDV RNA by qRT‐PCR in oropharyngeal fluid (OPF; blue), blood (red), oral swabs (green), nasal swabs (purple), and anal swabs (orange) in carriers (*n* = 9) and noncarriers (*n* = 31) from 7 to 28 or 35 days post‐challenge.

To explore the potential factors affecting the establishment of persistent FMDV infection in vaccinated cattle, a total of 40 cattle were included in this study. Among them, 20 were vaccinated with four different doses of serotype O FMDV VLPs monovalent vaccine, and the other 20 were vaccinated with four different doses of serotype A FMDV VLPs monovalent vaccine, with five cattle in each dose group. Homologous challenge was conducted on 35 dpv. During the 35‐day continuous sampling period after the challenge, the FMDV *3D* RNA loads in OPF and blood, as well as in nasal, oral, and anal swab samples from all animals, were monitored to evaluate infection dynamics and viral persistence (Figure 1A). Among the 40 vaccinated cattle examined, only two individuals developed vesicular lesions on the upper and lower gums at 3 dpc. These lesions resolved at 5 dpc, and these particular animals did not establish a persistent infection (Table 1), indicating that systemic generalization of infection does not affect the clinical outcome of FMDV infection in vaccinated cattle. FMDV *3D* RNA was consistently detected in OPF from 9 (22.5%) cattle throughout the sampling period, including 4 serotype O and 5 serotype A individuals, which were defined as FMDV carriers (Table 1). In contrast, the remaining 31 (77.5%) cattle showed nearly complete clearance of viral RNA in OPF by 28 dpc, with all animals testing negative by 35 dpc, defining them as noncarriers (Figure 1B). In addition, except for the occasional detection of very low levels of viral RNA in oral and nasal swabs from carriers, viral RNA in blood, nasal swabs, oral swabs, and anal swabs was largely undetectable from almost all animals after 14 dpc (Figure 1A, B). These results are consistent with previous reports [5, 9, 10], indicating that an FMDV persistent infection model was successfully established in vaccinated cattle in this study.

**Table 1:** Clinical symptoms and outcomes of vaccinated cattle after challenge.

Group (μg)	Animal ID	3 dpc	5 dpc	7 dpc	10 dpc	Carrier state
A‐12.5	05–15	‐	‐	‐	‐	Non‐carrier
	03–25	‐	‐	‐	‐	Non‐carrier
	372	‐	‐	‐	‐	Non‐carrier
	05–17	‐	‐	‐	‐	Non‐carrier
	05–14	Upper gums	‐	‐	‐	Non‐carrier

A‐25	99	‐	‐	‐	‐	Non‐carrier
	03–30	‐	‐	‐	‐	Carrier
	03–33	‐	‐	‐	‐	Carrier
	03–21	‐	‐	‐	‐	Non‐carrier
	03–28	‐	‐	‐	‐	Non‐carrier

A‐50	03–26	‐	‐	‐	‐	Non‐carrier
	06–22	‐	‐	‐	‐	Carrier
	98	‐	‐	‐	‐	Non‐carrier
	100	‐	‐	‐	‐	Carrier
	05–16	‐	‐	‐	‐	Non‐carrier

A‐100	03–35	‐	‐	‐	‐	Non‐carrier
	03–23	‐	‐	‐	‐	Non‐carrier
	91	‐	‐	‐	‐	Non‐carrier
	92	‐	‐	‐	‐	Carrier
	97	‐	‐	‐	‐	Non‐carrier

O‐12.5	93	‐	‐	‐	‐	Non‐carrier
	95	‐	‐	‐	‐	Non‐carrier
	94	‐	‐	‐	‐	Non‐carrier
	03–29	‐	‐	‐	‐	Non‐carrier
	03–36	‐	‐	‐	‐	Non‐carrier

O‐25	03–31	‐	‐	‐	‐	Non‐carrier
	03–32	‐	‐	‐	‐	Non‐carrier
	03–40	‐	‐	‐	‐	Carrier
	05–12	‐	‐	‐	‐	Non‐carrier
	03–37	‐	‐	‐	‐	Non‐carrier

O‐50	03–27	‐	‐	‐	‐	Non‐carrier
	06–01	Upper and lower gums	‐	‐	‐	Non‐carrier
	06–35	‐	‐	‐	‐	Non‐carrier
	06–39	‐	‐	‐	‐	Carrier
	06–40	‐	‐	‐	‐	Carrier

O‐100	05–4	‐	‐	‐	‐	Non‐carrier
	06–30	‐	‐	‐	‐	Non‐carrier
	06–31	‐	‐	‐	‐	Non‐carrier
	06–37	‐	‐	‐	‐	Carrier
	06–38	‐	‐	‐	‐	Non‐carrier

*Note*: ‐, absence of clinical symptoms.

Abbreviation: dpc, days post challenge.

To assess the efficacy of viral inoculation, four nonvaccinated cattle (two for each serotype) were included. The four nonvaccinated cattle exhibited varying degrees of vesicular lesions on both the upper and lower gums, as well as on the hooves of the forelimbs and hindlimbs postchallenge (Table S1). Only one of the nonvaccinated cattle exhibited trace levels of viral RNA in the OPF at 35 dpc, with undetectable viral RNA in the blood, as well as nasal, oral, and anal swabs by 14 dpc or 21 dpc (Figure S2). These findings suggest that vaccination does not significantly influence the establishment of persistent FMDV infection, as both vaccinated and nonvaccinated cattle demonstrated a comparable incidence of carrier state. Investigating the effects of viral serotypes and vaccine doses on viral persistence within the context of vaccinated status could provide theoretical insights for guiding FMD prevention and control strategies.

### 3.2. Correlation of Viral Serotypes or Vaccine Doses With FMDV Persistence

**Figure 2:**
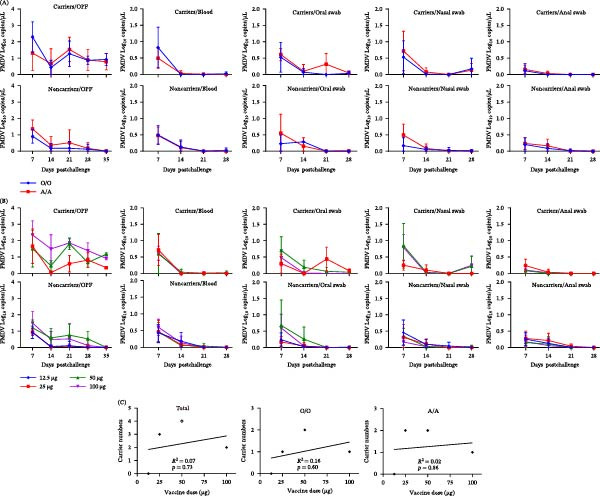
FMDV infection dynamics under different serotypes and vaccine doses. Comparison of FMDV RNA load by qRT‐PCR in OPF, blood, oral swabs, nasal swabs, and anal swabs in carriers and noncarriers from 7 to 28 or 35 days post‐challenge between serotype A and O (A) and among four vaccine doses (B). (C) The correlation between different serotypes or vaccine doses and carrier numbers. The two‐sided *p*‐values were determined by Pearson’s correlation test. O/O, vaccinated with serotype O and challenged with serotype O; A/A, vaccinated with serotype A and challenged with serotype A.

**Figure 3:**
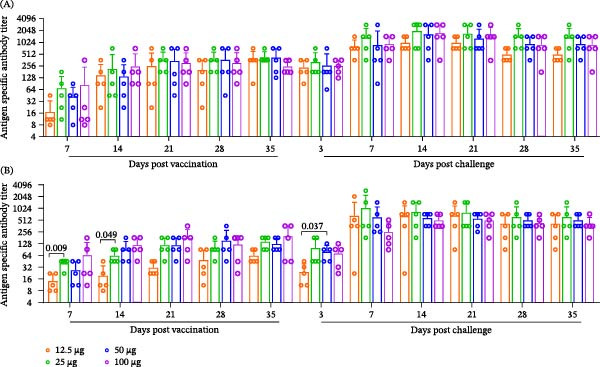
The dynamics of antigen‐specific antibody titer induced by four vaccine doses postvaccination and postchallenge. The titers of antigen‐specific antibody induced by four vaccine doses after vaccination and challenge in cattle vaccinated with serotype O vaccine and challenged with serotype O virus O/BY/2010 (*n* = 20) (A), and cattle vaccinated with serotype A vaccine and challenged with serotype A virus A/WH‐09 (*n* = 20) (B). O/O, vaccinated with serotype O and challenged with serotype O; A/A, vaccinated with serotype A and challenged with serotype A.

To assess the association between vaccine doses or viral serotypes and persistent FMDV infection, we compared viral shedding dynamics in carriers or noncarriers across different serotype and dose groups. The results indicated that either carriers or noncarriers vaccinated with type A or type O vaccines showed comparable viral shedding patterns postchallenge (Figure 2A), suggesting that serotype differences did not result in distinct shedding profiles. Likewise, carriers or noncarriers in the different vaccine‐dose groups also exhibited similar shedding patterns after challenge (Figure 3B). Notably, none of the cattle receiving the lowest dose of either serotype O or serotype A vaccines developed a carrier state, and noncarriers in the two lower‐dose groups cleared virus from OPF earlier (Figure 2B). In contrast, both carriers and noncarriers in the two high‐dose groups of both serotypes demonstrated higher viral loads than the two low‐dose groups, and viral RNA remained detectable for longer in OPF of noncarriers (Figure 2B; Figure S3A, B). Although differences in viral load and clearance kinetics were observed among dose groups, the correlation analysis revealed that the incidence of FMDV persistence was not significantly associated with either vaccine dose or viral serotype (Figure 2C).

### 3.3. Correlation of Systemic Antibody Levels With FMDV Persistence

**Figure 4:**
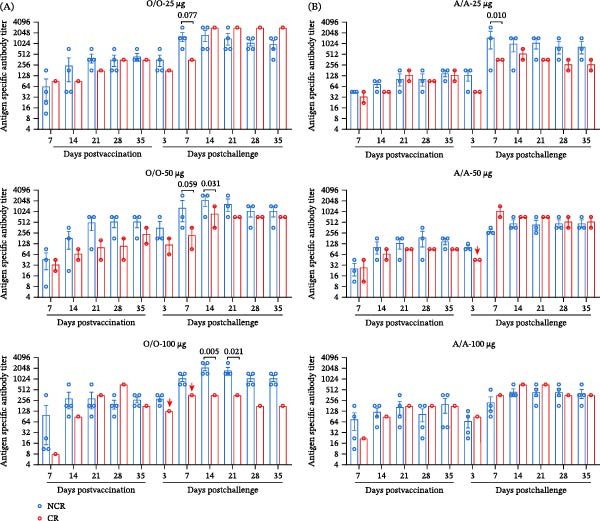
Antigen‐specific antibody titer profiles in carriers and noncarriers under different serotypes and vaccine doses postvaccination and postchallenge. The titers of antigen‐specific antibody postvaccination and postchallenge in cattle vaccinated with different doses of serotype O vaccine and challenged with O/BY/2010 (A) and cattle vaccinated with different doses of serotype A vaccine and challenged with A/WH‐09 (B). CRs, carriers; NCRs, noncarriers. O/O, vaccinated with serotype O and challenged with serotype O virus; A/A, vaccinated with serotype A and challenged with serotype A virus.

Next, we examined the association between antigen‐specific or NAb levels and the incidence of persistent FMDV infection in vaccinated cattle. During the postvaccination phase, no significant differences in antigen‐specific antibody levels were observed among cattle immunized with the four doses of the serotype O vaccine (Figure 3A). In contrast, cattle receiving the minimal dose of serotype A vaccine showed significantly lower antigen‐specific antibody levels at 7–14 dpv than those in the other dose groups, with this lower level persisting throughout subsequent vaccination periods (Figure 3B). However, following viral challenge, antigen‐specific antibody levels rose rapidly in all dose groups of both serotypes and were generally comparable across time points, except in the lowest‐dose serotype A group which showed lower levels at 3 dpc owing to weaker prechallenge baseline antibody responses (Figure 3A, B). Notably, irrespective of vaccine dose or serotype, antigen‐specific antibody titers showed no statistically significant differences between carriers and non‐carriers during the postvaccination period. In contrast, they were significantly lower in most carriers relative to noncarriers in the early posthallenge period (<14 dpc) (Figure 4A, B).

**Figure 5:**
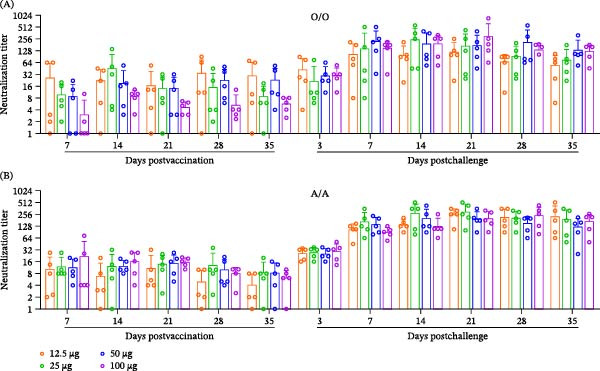
The dynamics of neutralizing antibody titer induced by four vaccine doses postvaccination and postchallenge. The titers of neutralizing antibody induced by four vaccine doses after vaccination and challenge in cattle vaccinated with serotype O vaccine and challenged with serotype O virus O/BY/2010 (*n* = 20) (A), and cattle vaccinated with serotype A vaccine and challenged with serotype A virus A/WH‐09 (*n* = 20) (B). O/O, vaccinated with serotype O and challenged with serotype O; A/A, vaccinated with serotype A and challenged with serotype A.

**Figure 6:**
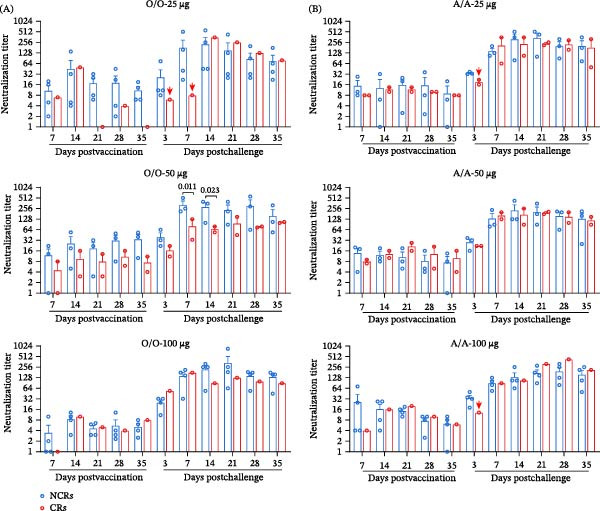
Neutralizing antibody titer profiles in carriers and noncarriers under different serotypes and vaccine doses postvaccination and postchallenge. The titers of neutralizing antibody postvaccination and postchallenge in cattle vaccinated with different doses of serotype O vaccine and challenged with O/BY/2010 (A) and cattle vaccinated with different doses of serotype A vaccine and challenged with A/WH‐09 (B). CRs, carriers; NCRs, noncarriers. O/O, vaccinated with serotype O and challenged with serotype O virus; A/A, vaccinated with serotype A and challenged with serotype A virus.

NAbs are essential for conferring protective defense against FMDV infection. Monitoring of NAb kinetics showed no significant differences across four dose groups of either serotype after vaccination or after challenge (Figure 5A, B). Consistent with the dynamics of antigen‐specific antibodies, a subset of carrier individuals also showed significantly lower NAb titers than noncarriers during the early postchallenge period, irrespective of vaccine doses or serotypes (Figure 6A, B). These findings indicate that, in comparison to noncarriers, carriers exhibit a delayed antibody response following viral exposure, which potentially contributes to the establishment of persistent infection in the nasopharynx.

### 3.4. Tissue‐Specific Viral Distribution in Carriers

**Figure 7:**
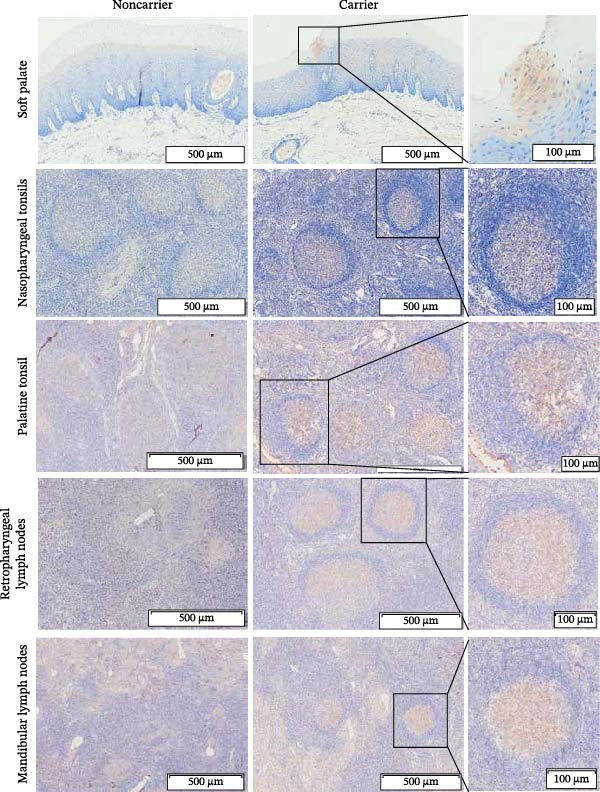
Representative IHC images of FMDV‐positive tissues. IHC analysis was conducted on tissues confirmed to be positive for FMDV RNA, with noncarrier tissues serving as negative controls. Increased germinal center numbers within the nasopharyngeal‐associated lymphoid and tonsil tissues were observed in carriers. The scale bar represents 500 μm for the main images and 100 μm for the magnified insets.

To determine the tissue‐specific localization of FMDV in carriers, we first quantified viral RNA loads in different anatomical sites using real‐time PCR. Among 22 anatomical sites, a higher incidence of viral RNA positivity was observed in samples from the nasopharynx, soft palate, and adjacent lymph nodes and tonsils of carriers (Table 2). Tissues with detectable viral RNA were subsequently analyzed by IHC using a monoclonal antibody against intact FMDV 146S virions. The results showed that in the soft palate, the virus exhibited a microfocal distribution pattern, with viral particles localized in the superficial epithelial cells. In addition, markedly increased numbers of germinal center structures were observed in the lymph nodes and tonsils of carriers (Figure 7), suggesting persistent antigenic stimulation at these sites accompanied by sustained local humoral immune activation.

**Table 2:** Tissue distribution of FMDV RNA during persistent phase of infection in FMDV carriers.

Strain	Carriers				Noncarriers			
	O/BY/2010		A/WH09		O/BY/2010		A/WH09	
Animal ID	0637	0639	92	100	0630	0631	0514	0517
Vaccine doses (µg)	100	50	100	50	100	100	12.5	12.5
Route of inoculation	V/I	V/I	V/I	V/I	V/I	V/I	V/I	V/I
Necropsy performed at dpc	35	35	35	35	35	35	35	35
Probang Log_10_ GCN/µL	1.22	1.08	1.13	0.84	neg	neg	neg	neg
Tissue sample (Log_10_ GCN/µL)								
Oral cavity/oropharynx								
Tongue	neg	0.15	1.11	neg	neg	0.17	neg	neg
Dental pad	neg	NE	neg	neg	neg	neg	NE	NE
Nasopharynx/larynx								
Soft palate	0.99	NE	1.06	0.15	neg	NE	0.10	neg
Nasopharynx	0.35	1.82	1.03	0.05	neg	neg	neg	neg
Nasopharyngeal tonsil	0.31	0.86	1.41	neg	neg	NE	0.25	NE
Palatine tonsil	neg	0.08	1.04	neg	neg	0.1	neg	NE
Lingual tonsil	neg	0.81	0.25	0.42	neg	neg	neg	neg
Epiglotis	1.08	1.65	1.30	neg	neg	neg	neg	neg
Alimentary system								
Rumen	neg	0.16	0.06	0.04	neg	NE	neg	neg
Reticulum	0.21	NE	1.07	0.14	neg	0.22	neg	0.07
Omasum	neg	NE	1.07	neg	neg	0.01	neg	0.06
Abomasum	neg	0.29	1.01	neg	neg	NE	neg	0.13
Dodecadactylon	NE	neg	neg	neg	neg	0.12	neg	neg
Jejunum	neg	neg	neg	neg	NE	neg	neg	neg
Ileum	neg	0.01	1.06	neg	neg	neg	neg	neg
Colon	neg	0.08	1.07	neg	neg	NE	neg	neg
Cecum	NE	neg	0.93	0.23	neg	NE	neg	neg
Rectum	neg	0.22	0.94	neg	neg	NE	neg	neg
Anus	0.02	NE	0.87	neg	NE	NE	NE	neg
Additional tissues								
Medial retropharyngeal LN	0.04	0.06	1.15	0.17	neg	neg	neg	neg
Submandibular LN	0.85	0.20	0.06	0.86	neg	0.47	neg	0.35
Coronary band	neg	0.34	neg	NE	neg	NE	NE	0.03

*Note:* The numbers in the table represent the log_10_ genome copy number (GCN) per milliliter of FMDV RNA in tissue or OPF samples. V/I, vaccinated and challenged by intradermolingual inoculation.

Abbreviations: dpc, days post challenge; ID, identifier; LN, lymph node; NE, not evaluated; neg, negative.

## 4. Discussion

Persistent FMDV infection in ruminants represents a major barrier to disease control and eradication in endemic regions. Although vaccination effectively reduces the size of susceptible populations, FMDV can still establish persistent infection in vaccinated cattle, leading to covert viral transmission. This not only increases the difficulty of FMD control but also seriously hinders its eradication. Previous studies have extensively investigated the clinical characteristics of FMDV carriers and the potential mechanisms underlying carrier‐state formation; however, differences in study design, particularly in animal species and strain types, have led to various distinct findings. The relationship between the establishment of persistent infection and viral serotype or vaccine dose remains unclear.

In this study, a persistent infection model was experimentally established in cattle vaccinated with different doses of serotype O and serotype A vaccines. By comparing viral shedding kinetics, antigen‐specific antibody levels, and NAb responses under different serotype and vaccine‐dose groups, we systematically evaluated the effects of viral serotype and vaccine dose on the incidence of persistent FMDV infection. Comparison of postchallenge viral shedding dynamics showed that, regardless of whether animals became carriers, cattle vaccinated with serotype A or O vaccines exhibited broadly similar shedding patterns. Although the vaccine dose had some effect on viral load and shedding duration, it did not significantly affect the incidence of persistent infection. Thus, in our study, neither FMDV serotype nor vaccine dose was distinctly associated with the establishment of persistent infection.

The strength of the humoral immune response induced by FMD vaccines is closely related to vaccine‐mediated protection. In this study, although carriers and noncarriers did not differ significantly in antigen‐specific or NAb levels before challenge, some carriers exhibited significantly lower serum antigen‐specific and NAb levels than noncarriers during the early postchallenge period (<14 days), regardless of serotype or vaccine dose; however, these differences disappeared after 14 dpc. This finding suggests that the timing of early humoral response initiation, rather than steady‐state antibody levels, may be closely linked to the establishment of persistent infection. Previous studies have shown that early antibody responses induced by FMDV are positively correlated with effective induction of Th1 responses and cytotoxic T lymphocyte (CTL) responses [28, 30–32]. Our recent research revealed that carriers immunized with inactivated FMD vaccines exhibited significantly lower frequencies of IFN‐γ‐producing CD4^+^ and CD8^+^ T lymphocytes than noncarriers during the early postchallenge phases [33]. Consequently, future studies should further elucidate the differences in cell‐mediated immune responses between carriers and noncarriers, as well as the mechanisms underlying these differences. In addition, vaccine designs or intervention strategies aimed at enhancing Th1‐biased immune responses and robust CD8^+^ T‐cell activity potentially mitigate the risk of persistent FMDV infections.

Effective viral clearance may also depend on the host susceptibility or disease resistance. In our persistent infection model using local cattle‐yak (*Bos grunniens* × *Bos taurus*), the carrier rate was 22.5%, substantially lower than the 50%–100% reported in Holstein (*Bos taurus*) cattle and buffaloes [5, 10, 23, 34, 35]. Notably, one low‐dose serotype O recipient (0329) failed to mount adequate NAb responses postvaccination (≤1:2) and postchallenge (1:6–1:22) yet cleared the virus without symptoms. Comparative transcriptomic analyses of lung tissues from multiple bovine breeds have revealed that yaks and Tibetan cattle (*Bos mutus*) exhibit higher basal activity in innate immune pathways [36]. Another study reported that *Bos indicus* breeds exhibit stronger mitochondrial and antiviral responses during acute FMDV infection [37], with earlier induction of NAbs and IFN‐γ responses, as well as lower viral loads in nasal and oral secretions. These findings suggest that the host genetic background or breed differences may influence viral clearance efficiency and the probability of persistent infection by modulating the threshold for innate immune activation and the magnitude of adaptive immune responses. Recent whole‐genome resequencing work from our laboratory further showed that variants associated with the FMDV carrier state are mainly distributed in genomic regions related to olfactory perception, cell development and structural maintenance, transcriptional and translational regulation, signal transduction, metabolic homeostasis, stress tolerance, and immune cell regulation. Although no single deterministic mutation was identified across carriers, different combinations of variants may collectively influence innate immune regulation and the strength of adaptive immune activation, thereby conferring interindividual differences in antiviral capacity [38].

Using qRT‐PCR and IHC, our research confirmed the presence of the FMDV genome and virions in various anatomical sites, including the soft palate, nasopharynx, and their adjacent tonsils and lymph nodes of carriers [9, 39, 40]. Initial infection typically begins in the soft palate epithelium before spreading to regional lymph nodes [41]. Consequently, effective control of persistent FMDV infection will require strategies targeting mucosal barriers to block early replication and prevent persistence. Persistent infection generally implies prolonged antigenic stimulation, which may sustain germinal center responses. In a chronic LCMV infection model, persistently active germinal center B‐cell responses and continued antibody production were observed [42]. Consistent with this, our study also observed that germinal center structures were markedly increased in lymph nodes and tonsils surrounding the nasopharynx of carriers, suggesting that local secondary lymphoid tissues remain in a state of sustained antigen‐driven humoral activation. This persistently active B‐cell response may also explain why mucosal sIgA levels remain elevated for an extended period after infection in carriers [33, 43–45].

Although we systematically compared the effects of serotypes and vaccine doses on the establishment of persistent infection, thereby providing theoretical support for disease prevention and control, we are fully aware of the limitations of this study. These include reduced statistical power due to small sample sizes. Constraints in laboratory capacity, together with eradication policies, have made it difficult to obtain a substantial number of samples from animals with persistent FMDV infection. In addition, we did not pay attention to the differences in immune responses between vaccinated and unvaccinated animals following the challenge. The differences among cattle breeds or between vaccine formulations were also not systematically evaluated. Further research involving clinical and molecular immunological features generated by a broader range of FMDV strains, vaccine preparations, and cattle breeds is warranted to elucidate.

## 5. Conclusion

In conclusion, our findings indicate no association of serotypes and vaccine doses with FMDV persistence. Intriguingly, a subset of carriers exhibited markedly lower antibody levels in the early postchallenge phase. The persisting virus was mainly located in the nasopharynx, soft palate, and adjacent lymph nodes and tonsils of the carriers. An increased number of germinal centers within nasopharyngeal‐associated lymph nodes and tonsils of carriers potentially addressed the knowledge gaps that elevated sIgA levels in nasopharyngeal secretions of carriers sustained for an extended period following viral infection. Strategies aimed at enhancing early immune activation, eliciting robust cell‐mediated responses, and providing effective mucosal protection may hold promise for reducing the incidence of FMDV persistence in ruminants.

## Author Contributions

Conceptualization: Huichen Guo, Shiqi Sun, and Zhihui Zhang. Supervision: Huichen Guo, Shiqi Sun, and Yijing Li. Project administration: Huichen Guo and Shiqi Sun. Sampling: Shuanghui Yin, Zhidong Teng, Suyu Mu, and Yaozhong Ding. Investigation: Zhihui Zhang, Zhidong Teng, Sumin Wei, Suyu Mu, Jinwei Huang, Shuang Wang, Yun Zhang, Shuanghui Yin, and Guoliang Huang. Data analysis and visualization, writing – original draft preparation: Zhihui Zhang. Writing – review and editing: Huichen Guo, Shiqi Sun, and Zhihui Zhang.

## Funding

This work was supported by the National Natural Science Foundation of China (Grants 32473012 and 32301127), the Key R&D Program of Ningxia province (Grant 2024BBF02017), the National Key R&D Program of China (Grant 2021YFD1800303), and the Science and Technology program of Gansu Province (Grants 24CXNA030, 25JRRA1086, 25JRRA1089, and 25YFNA014).

## Disclosure

All authors read, reviewed, and approved the submitted manuscript.

## Conflicts of Interest

The authors declare no conflicts of interest.

## Supporting Information

Additional supporting information can be found online in the Supporting Information section.

## Supporting information


**Supporting Information** Figure S1: Characterization of FMDV‐VLPs. SDA‐PAGE and western blotting of purified proteins and VLPs for serotype O (A) and A (B). M: Marker; Lane 1: His‐SUMO tagged‐capsid proteins of FMDV; Lane 2: Enzyme‐digested and self‐assembled capsid proteins without His‐SUMO tag. DLS measured the hydrated particle size of VLPs for serotype O (C) and A (D). Figure S2: Infection dynamics of non‐vaccinated cattle. Detection of FMDV RNA by qRT‐PCR in OPF, blood, oral swabs, nasal swabs, and anal swabs from 7 to 28 or 35 days post‐challenge. Figure S3: FMDV infection dynamics under different serotypes and vaccine doses. Comparison of FMDV RNA load in OPF from carriers (*n* = 4) and noncarriers (*n* = 16) administrated with different doses of serotype O vaccine (A), as well as carriers (*n* = 4) and noncarriers (*n* = 16) administrated with different doses of serotype A vaccine (B) from 7 to 35 days post‐challenge. Table S1: Clinical symptoms of non‐vaccinated cattle after challenge.

## Data Availability

The data that support the findings of this study are available from the corresponding author upon reasonable request.
