# Supplementary material for: Foot‐and‐Mouth Disease Virus Persistence Divergence Within Serotypes and Vaccine Doses in Vaccinated Cattle
Source: Transbound Emerg Dis. 2026 Jun 26;2026:5568178. doi: 10.1155/tbed/5568178 (PMC13305672; doi:10.1155/tbed/5568178)
Supplement: Supplementary file 1 — Supporting Information Figure S1: Characterization of FMDV‐VLPs. SDA‐PAGE and western blotting of purified proteins and VLPs for serotype O (A) and A (B). M: Marker; Lane 1: His‐SUMO tagged‐capsid proteins of FMDV; Lane 2: Enzyme‐digested and self‐assembled capsid proteins without His‐SUMO tag. DLS measured the hydrated particle size of VLPs for serotype O (C) and A (D). Figure S2: Infection dynamics of non‐vaccinated cattle. Detection of FMDV RNA by qRT‐PCR in OPF, blood, oral swabs, nasal swabs, and anal swabs from 7 to 28 or 35 days post‐challenge. Figure S3: FMDV infection dynamics under different serotypes and vaccine doses. Comparison of FMDV RNA load in OPF from carriers (n = 4) and noncarriers (n = 16) administrated with different doses of serotype O vaccine (A), as well as carriers (n = 4) and noncarriers (n = 16) administrated with different doses of serotype A vaccine (B) from 7 to 35 days post‐challenge. Table S1: Clinical symptoms of non‐vaccinated cattle after challenge. [file TBED-2026-5568178-s001.docx]

**Preparation and characterization of FMD VLPs**

The purified structural proteins after induced expression and the assembled VLPs after enzymatic cleavage were analyzed by SDS-PAGE and Western blot. The results showed that, both before cleavage and after assembly, the molecular weights of the three structural proteins of serotype A and serotype O FMDV were consistent with the predicted values and could be recognized by specific antibodies (Figure S1A-B), indicating that the three structural proteins of both FMDV serotypes were successfully purified. Hydrodynamic size analysis showed that the assembled serotype O and serotype A samples had mean hydrodynamic diameters of approximately 37.84 nm and 32.6 nm (Figure S1C-D), respectively, indicating that VLPs of both serotypes were successfully assembled.


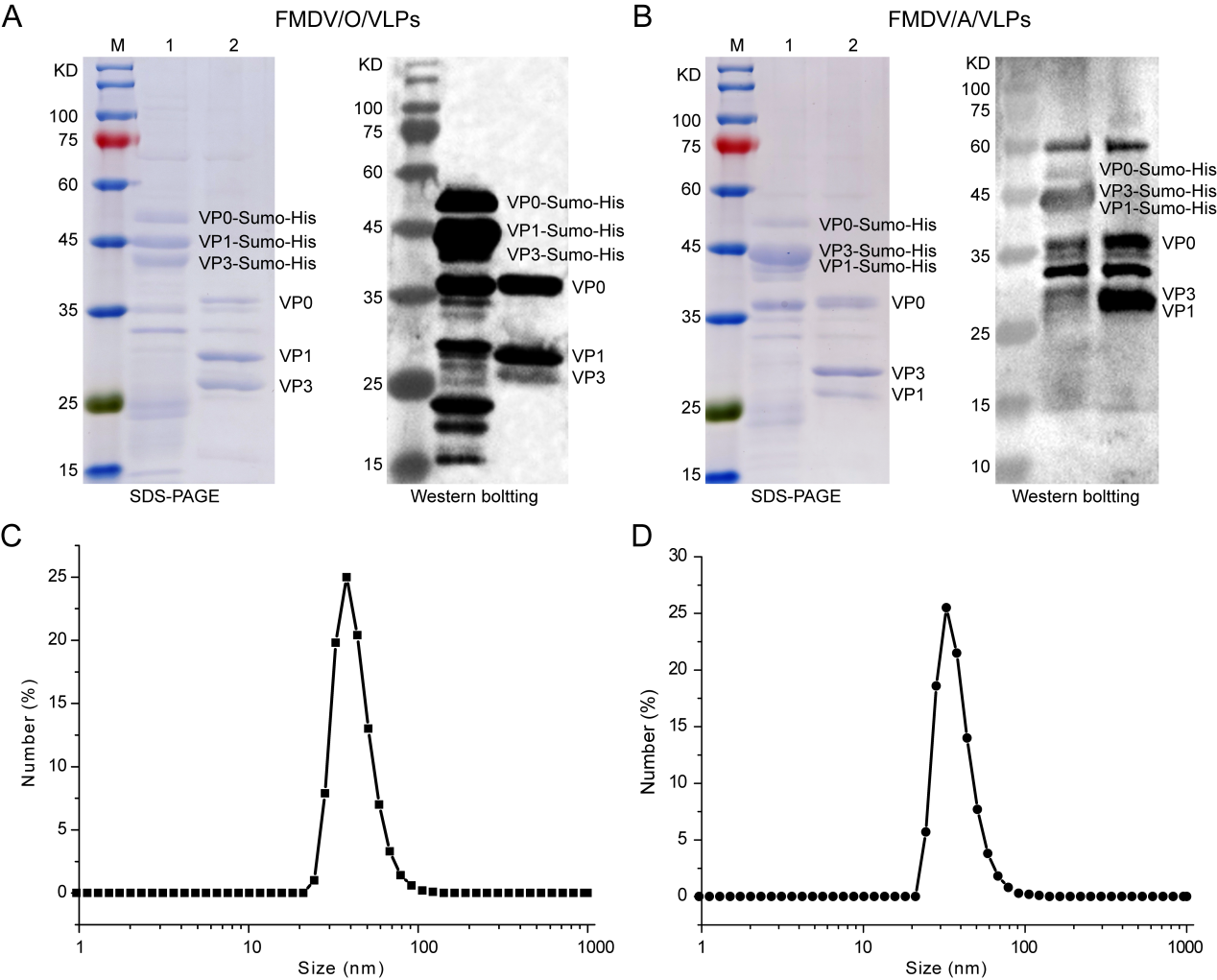


**Figure S1. Characterization of FMDV-VLPs.** SDA-PAGE and western blotting of purified proteins and VLPs for serotype O (A) and A (B). M: Marker; Lane 1: His-SUMO tagged-capsid proteins of FMDV; Lane 2: Enzyme-digested and self-assembled capsid proteins without His-SUMO tag. DLS measured the hydrated particle size of VLPs for serotype O (C) and A (D).


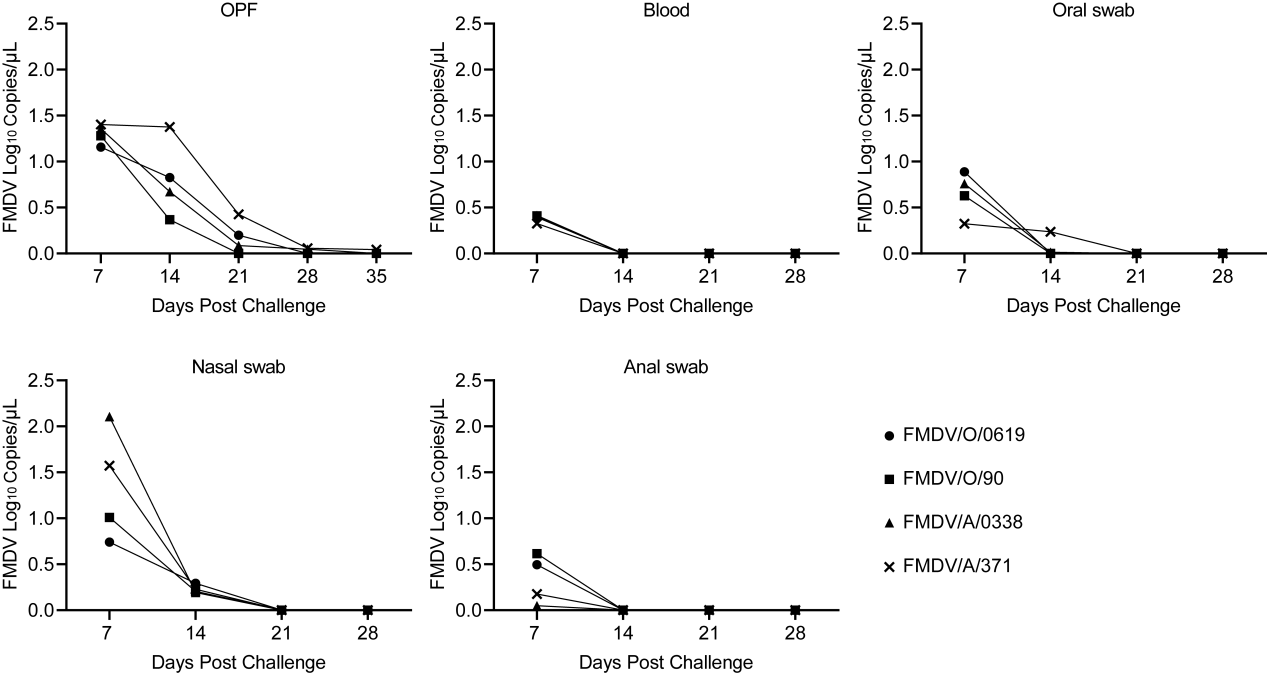


**Figure S2. Infection dynamics of non-vaccinated cattle.** Detection of FMDV RNA by qRT-PCR in OPF, blood, oral swabs, nasal swabs, and anal swabs from 7 to 28 or 35 days post-challenge.


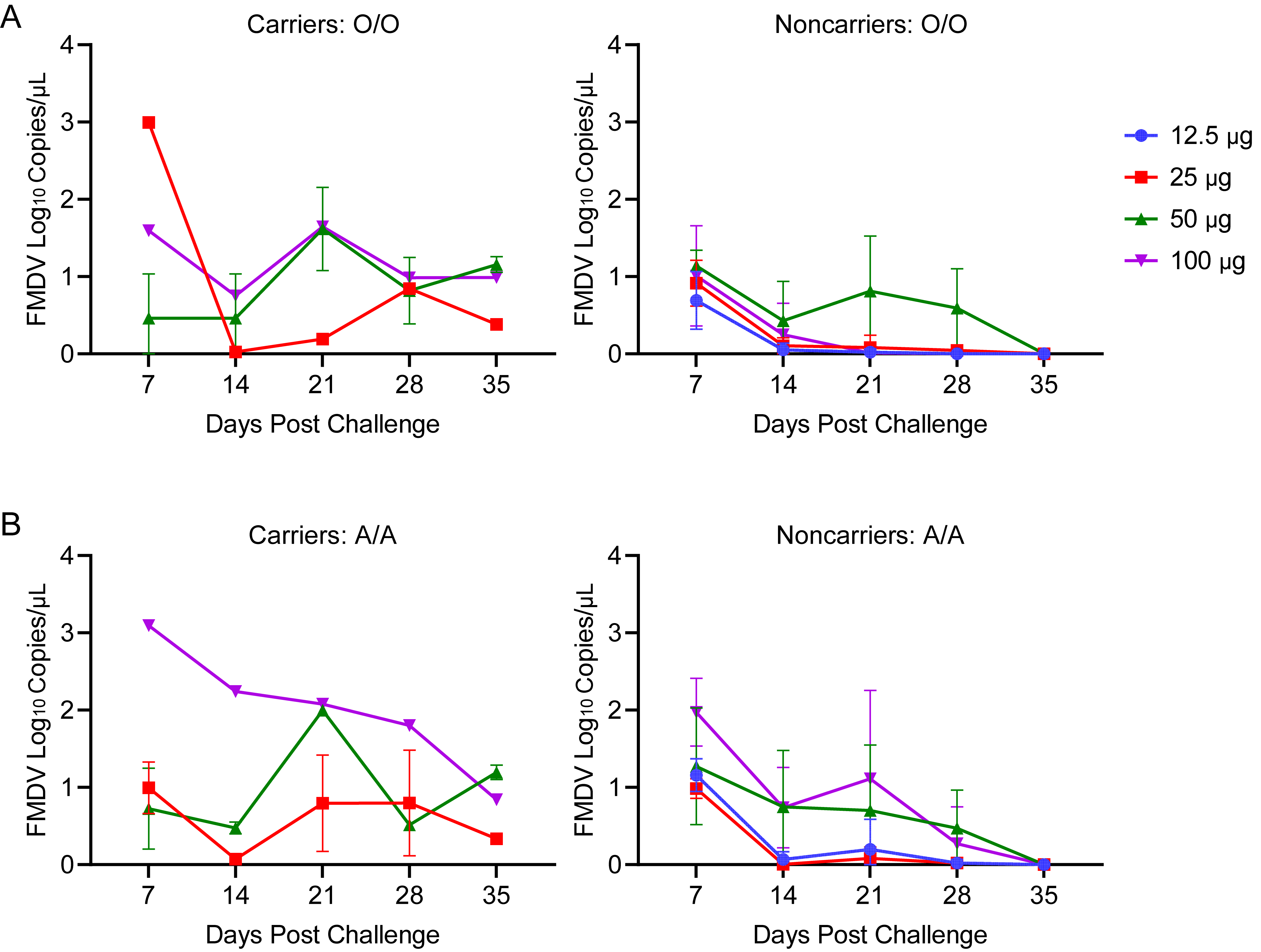


**Figure S3. FMDV infection dynamics under different serotypes and vaccine doses.** Comparison of FMDV RNA load in OPF from carriers (*n* = 4) and noncarriers (*n* = 16) administrated with different doses of serotype O vaccine (A), as well as carriers (*n* = 4) and noncarriers (*n* = 16) administrated with different doses of serotype A vaccine (B) from 7 to 35 days post-challenge.

**Table S1. Clinical symptoms of non-vaccinated cattle after challenge**

| **Group** | **Animal ID** | 3 dpc | 5 dpc | 7 dpc | 10 dpc |
| --- | --- | --- | --- | --- | --- |
| FMDV/A | 03-38 | upper and lower gums | right front hoof | - | - |
|  | 371 | upper gums |  | right front hoof | - |
| FMDV/O | 06-19 | lower gums | left and right front hooves | left and right front, left hind foot | - |
|  | 90 | upper gums | left and right front hooves | left and right front hooves, left hind foot | - |
